# Intracystic papillary carcinoma of the breast in males: Three case reports

**DOI:** 10.1097/MD.0000000000020278

**Published:** 2020-06-19

**Authors:** Hua Luo, Kexin Meng, Junling He, Zujian Hu, Ouou Yang, Tian Lan, Kunlun Su, Huifen Yang, Chenni Zhan, Haibin Xu

**Affiliations:** a Department of Breast Surgery, Hangzhou Hospital of Traditional Chinese Medicine; b Department of Thyroid Breast Surgery, Zhejiang Provincial People's Hospital, People's Hospital of Hangzhou Medical College, Hangzhou, Zhejiang Province, China.

**Keywords:** contrast-enhanced ultrasonography, intracystic papillary carcinoma, males

## Abstract

**Rationale:**

Intracystic papillary breast carcinoma is extremely rare in males with a favorable prognosis. Clinical and mammographic manifestations of IPC are not specific, and no consensus has been reached on its management.

**Patient concerns:**

Three cases of IPC of the breast in male patients who underwent surgery are presented. In each patient, clinical manifestations, radiological appearance, surgical procedures, pathological diagnosis, and prognosis were investigated.

**Diagnosis:**

Ultrasonography showed a complex mass with cystic and nodular solid components in 2 patients and a solid hypoechoic mass in the other 1. Contrast-enhanced ultrasonography(CEUS) was performed for 1 patient demonstrated a solid component of the characteristic enhancement patterns. The final diagnosis of IPC was made after an excisional biopsy.

**Interventions:**

A mastectomy with sentinel lymph node mapping was carried out in 2 patients, and it was negative for metastatic disease. The third patient received a mastectomy without an investigation of the axillary lymph node status.

**Outcomes:**

All the patients are disease-free during a median follow-up of 67 months (range, 13–120) months.

**Lessons:**

It is difficult to diagnose IPC of the male breast before surgery, excisional biopsy is necessary. CEUS can be useful to diagnose IPC in male patients in the preoperative evaluation. Sentinel node biopsy may be considered in patients with IPC associated with DCIS or invasive carcinoma.

## 1 Introduction

Male breast cancer is a rare entity which constitutes only 0.6% of breast cancer.^[1]^ Intracystic papillary carcinoma (IPC) originates as a solitary tumor in a cystic and dilated duct. This breast cancer subtype with low incidence is commonly regarded as low-grade carcinoma. Male breast cancer patients with pathological diagnosis of intracystic papillary carcinoma are extremely rare, and its clinical characteristic is poorly understood. Here, we report 3 cases of IPC of the breast in male patients with detailed pathologic and radiologic records and review the literature to describe this disease fully. A presentation of the summary of all cases is exhibited in Table 1.

**Table 1:**
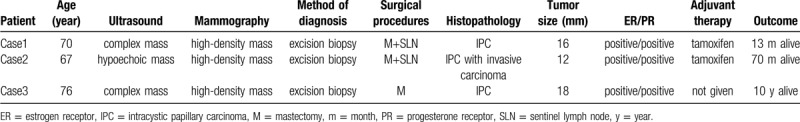
Review of the clinicopathological features of 3 intracystic papillary carcinoma in males.

## 2 Case presentation

### 2.1 Case 1

**Figure 1:**
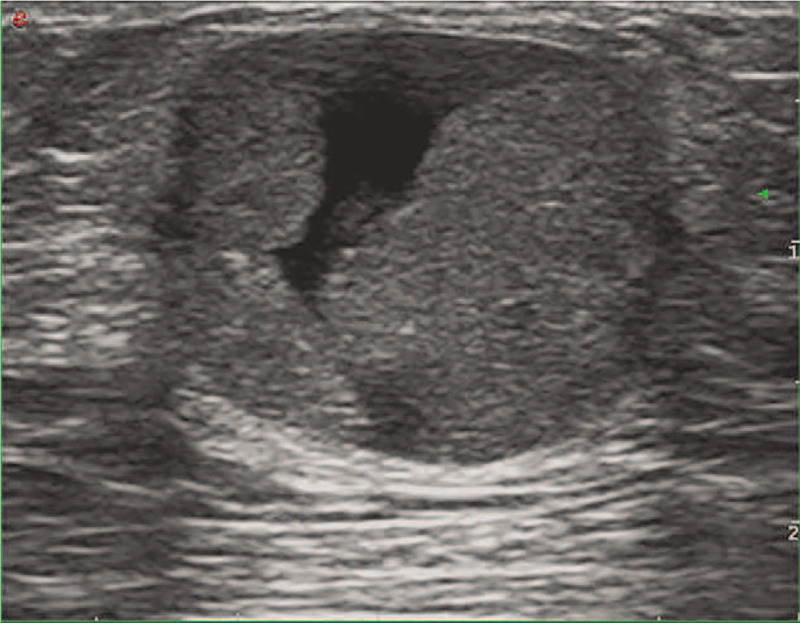
The long-axis sonogram of the left breast showed a regular shaped, well-defined complex mass with the cystic and solid hypoechoic mass in the retro areolar region.

A 70-year-old man presented with a painless mass in his left subareolar region 4 years ago. A regular examination was scheduled, and the tumor enlarged. He was then referred to our hospital for further investigation. He had a history of hypertension and no breast cancer family history. Physical examination revealed a 2 cm well-circumscribed, firm, and mobile mass in the left periareolar region. The nipple was not retracted. No sign of gynecomastia. Bilateral axillary lymph nodes were not palpable. Ultrasound (US) showed a regular shaped, well-defined complex mass measuring 25 × 20 × 15 mm with cystic and solid hypoechoic. This mass was in the left retro areolar region, with posterior acoustic enhancement (Fig. 1).

**Figure 2:**
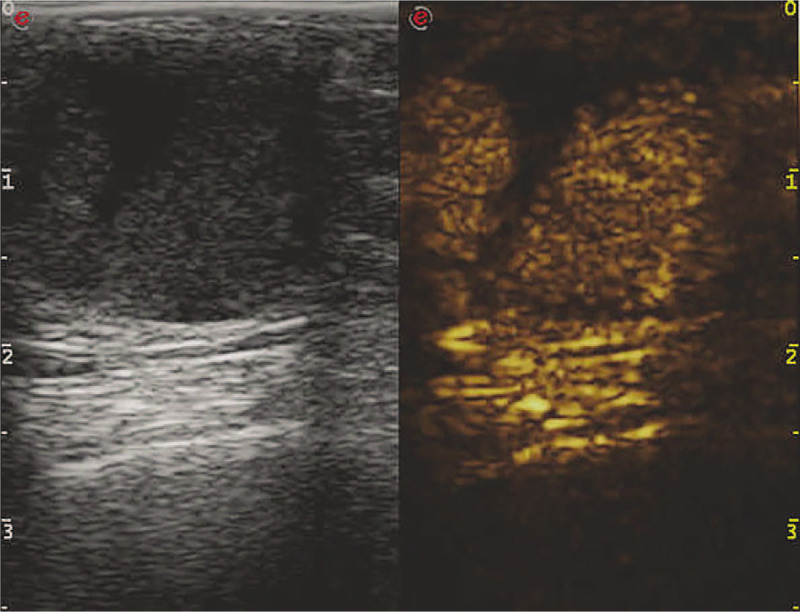
CEUS demonstrated a rapid wash-in, slow wash-out with heterogeneous hyperenhancement lesion (hyperenhancement with solid and perfusion defect with cystic components).

Contrast-enhanced ultrasonography (CEUS) demonstrated a solid component of the enhancement patterns (rapid wash-in, slow wash-out with heterogeneous hyperenhancement) and cystic component perfusion defect. The shape after enhancement was the same as that demonstrated on routine 2D gray-scale images with clear margins, regular shape, and penetrating bloodstream (Fig. 2). The lesion was classified as US-BI-RADS (Breast Imaging Reporting and Data System)4C.

**Figure 3:**
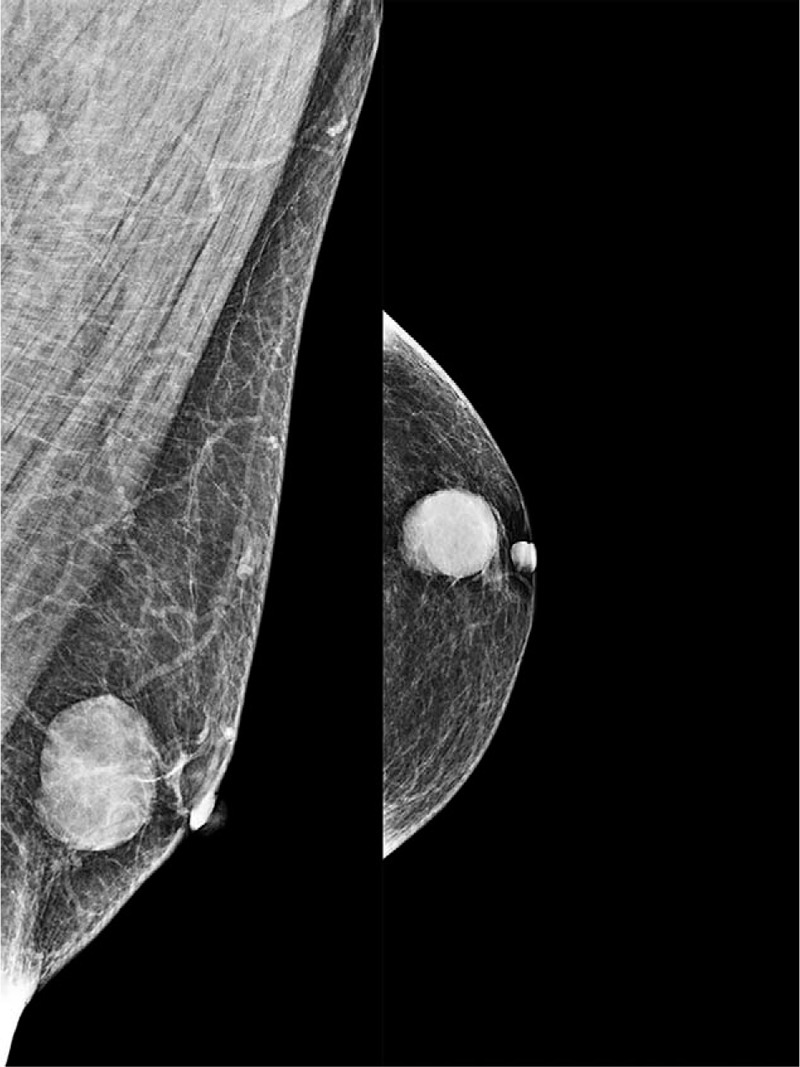
The mammogram showed a round, high-density mass with a regular but partially obscured margins beneath the nipple of the left breast.

Mammography showed a round, high-density mass with a regular but partially obscured margins, measuring approximately 22 × 21 mm. No spiculation or microcalcification was found. The lesion was classified as BI-RADS category 4A (Fig. 3).

The diagnosis of intracystic papillary carcinoma (IPC) was made after excisional biopsy, and the patient underwent simple mastectomy with axillary sentinel lymph node biopsy later. No positive axillary lymph node was detected.

Grossly, the size of the lesion was 1.6 x 1.5 cm. The substantial component was diagnosed as IPC. The carcinoma cells were cribriform arranged, solid, and mild atypia. On immunohistochemical staining, the cancer cells were strongly positive for estrogen receptor (90% of cells positive) and progesterone receptor (> 99% of cells positive), negative for human epidermal growth factor receptor 2 (HER-2), and the Ki-67 score was 10%. The patient is currently receiving adjuvant tamoxifen and without recurrence 13 months postoperatively.

### 2.2 Case 2

A 67-year-old-man presented with a painless lump in the right breast for 3 months. He had no nipple discharge and no familial history of breast carcinoma. The patient had a history of diabetes mellitus. Physical examination revealed a 1.5 cm in diameter, well-circumscribed, and firm mass in the right subareolar region. The tumor was fixed. The nipple was not retracted. Bilateral axillary lymph nodes were not palpable.

**Figure 4:**
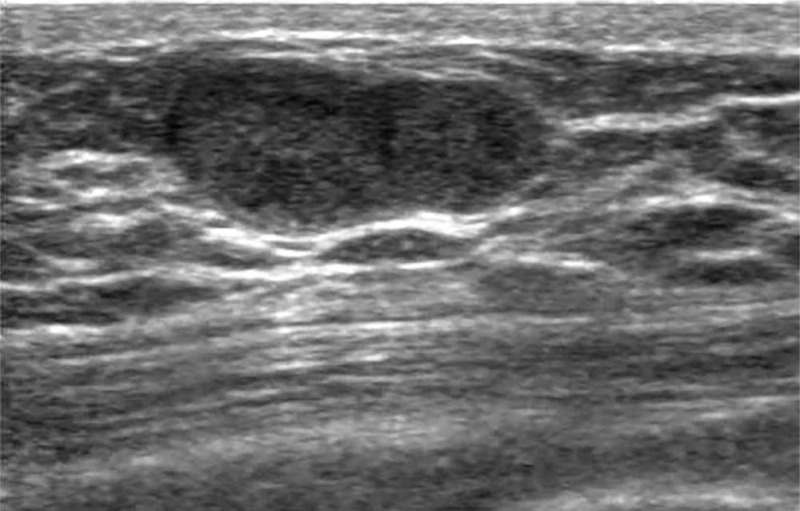
Ultrasonography demonstrated a regular shaped, well-defined hypoechoic mass in the subareolar region of the right breast.

**Figure 5:**
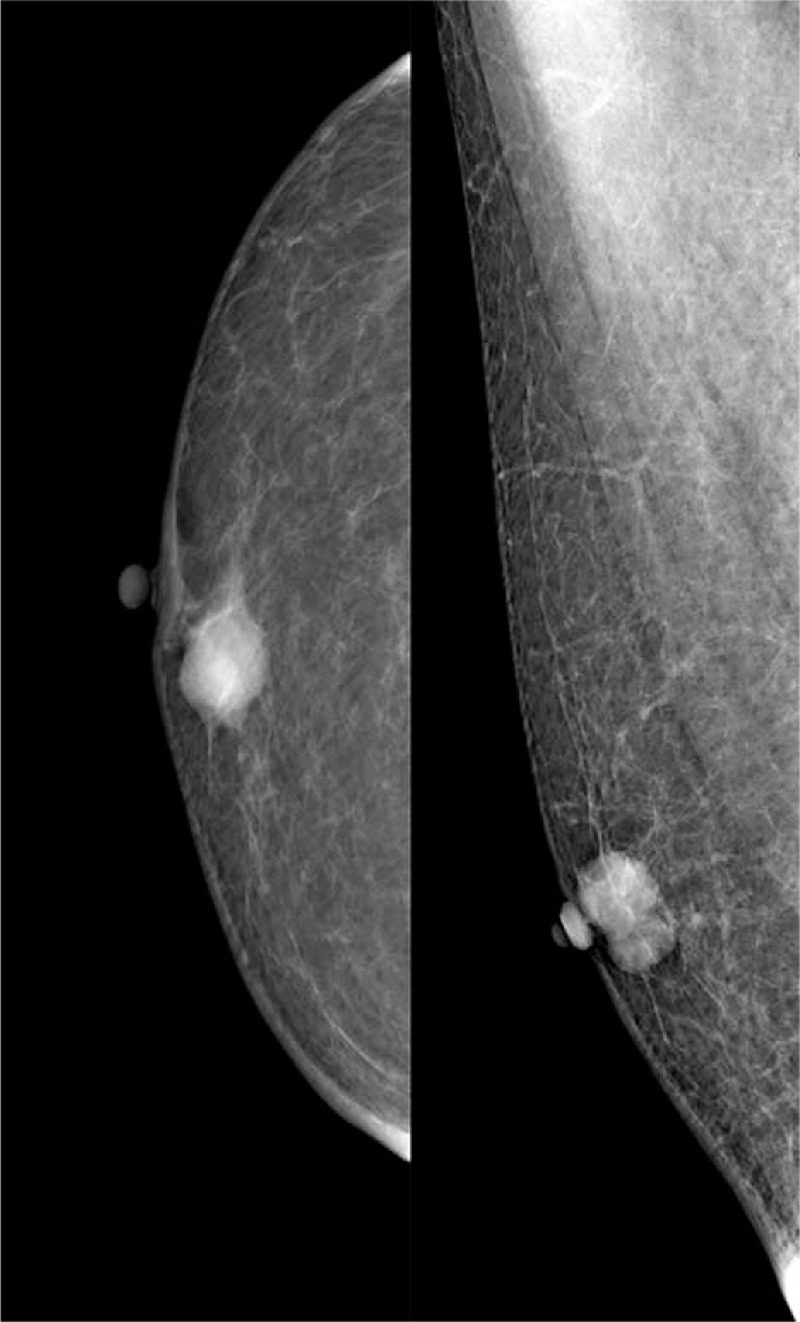
Mammography demonstrated a relatively distinct and dense mass without microcalcification or spiculation under the nipple of the right breast.

Ultrasonography demonstrated a regular shaped, well-defined hypoechoic mass measuring 12 × 10 × 8 mm located just below his right subareolar region (Fig. 4). Mammography showed a 17 × 15 mm, relatively distinct, and dense mass without microcalcifications or spiculations under the nipple of the right breast, which was classified as BI-RADS 4B (Fig. 5).

Excision biopsy reported an intracystic papillary carcinoma. Mastectomy with sentinel lymph node mapping was carried out, and it was negative for metastatic disease. The pathology of the specimen showed a 1.2 cm IPC with a small focus of invasive carcinoma without lymphovascular infiltration.

Immunohistochemistry showed that the tumor was positive for estrogen and progesterone receptors, negative for HER-2, and had a Ki-67 score of 35%. On immunohistochemical staining, myoepithelial cells with p63, calponin, and smooth muscle myosin were negative, supporting the presence of invasive carcinoma.

Tamoxifen 20 mg daily was started therapy, and no disease recurrence was reported 70 months postoperatively.

### 2.3 Case 3

**Figure 6:**
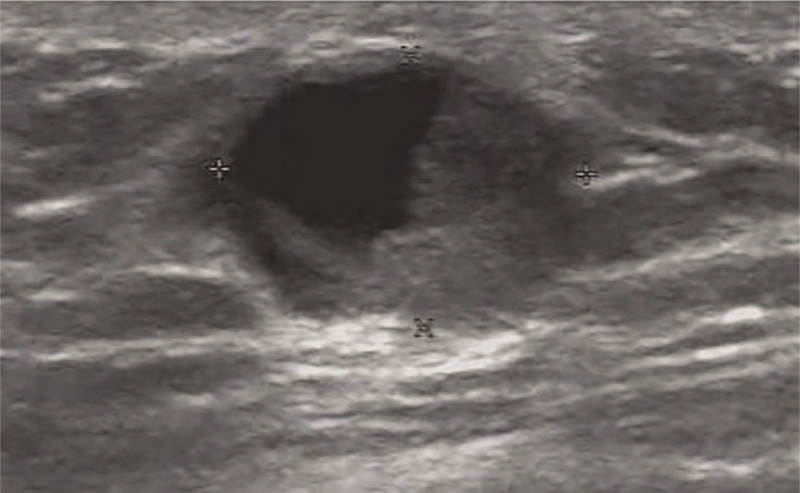
Ultrasound of the right breast demonstrated a well-circumscribed, complex mass.

A 76-year-old-man presented with a painless lump in the right breast for 3 weeks. He had a medical history of hypertension. Physical examination revealed a 1 cm mobile lump in the right breast with no palpable axillary lymphadenopathy and no other significant findings. Ultrasound of the right breast lump demonstrated a well-circumscribed, complex mass, measuring 14 × 13 × 10 mm. Color Doppler US showed no vascular flow within the solid component of the mass (Fig. 6).

**Figure 7:**
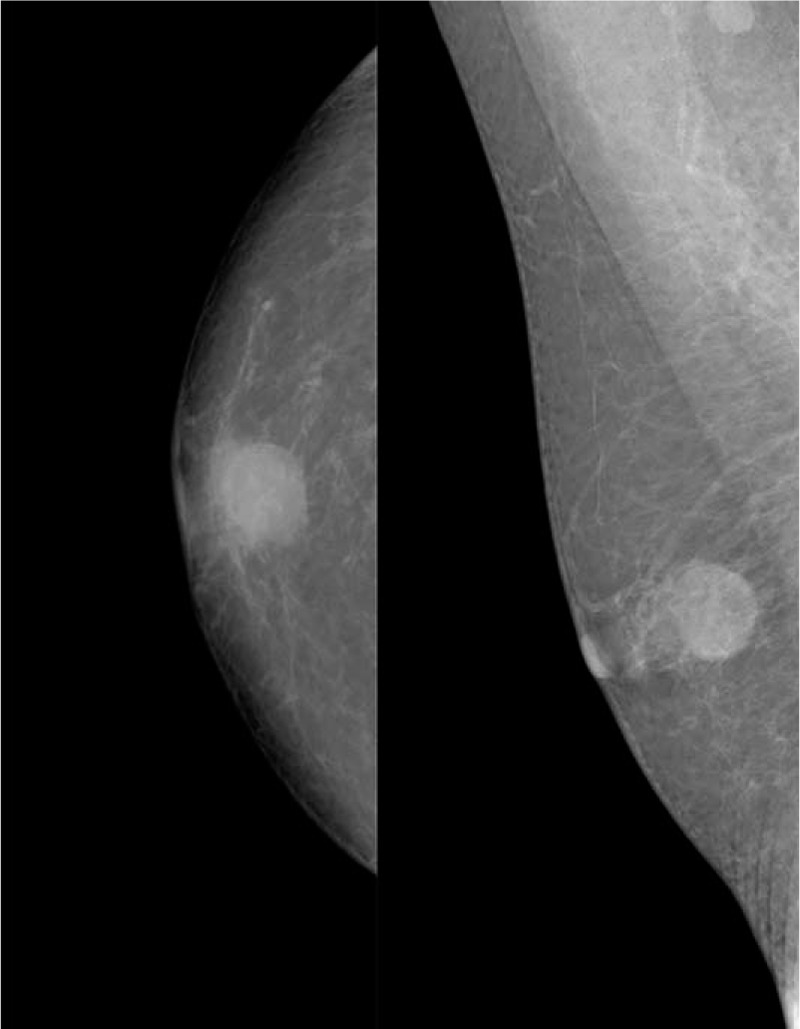
Mammography showed a partially obscured margins high-density mass beneath the nipple of the right breast.

Mammography demonstrated a 15 × 13 mm, well defined dense mass without microcalcification, and classified as BI-RADS 4C (Fig. 7).

The patient subsequently underwent lumpectomy of the right breast mass. Histological analysis revealed a 1.5 cm × 1.8 cm × 1.2 cm cyst lesion, and the lesion was comprised of a papillary and solid proliferation of atypical cells within the cyst. The final diagnosis confirmed IPC. On immunohistochemistry, the tumor was estrogen receptor-positive, and progesterone receptor-positive and Her-2 negative, and had a Ki-67 score of 60%. Then the patient underwent right mastectomy without any adjuvant treatment. There were no local recurrences during a follow-up of 10 years.

## 3 Discussion

Breast carcinoma in men is rare, accounting for 0.6% of all breast carcinomas.^[1]^ IPC is rare, and it represents approximately 1% of all breast cancers.^[2]^ IPC accounts for 0.5% to 2% of breast cancers in females.^[3,4]^ IPCs in male patients are rare, accounting for only 3.5% of 927 IPC cases.^[5]^

IPC develops predominantly in elderly patients. Patients with IPC tend to be older than other types of breast cancer. Tochika et al reported the mean age of IPC in males as 68.2 years.^[6]^ Yoshida, Mouri et al reported the mean age of the patients with IPC in males was 69 years (range 41–91).^[7]^

The median age of IPC patients was 69.5 years (range 27 years-99 years) in 917 cases of IPC.^[5]^

The age of patients ranged between 67 and 76 years (the mean age is 71 years) in our circumstances. It may mimic as gynecomastia; 1 of our cases described above had a history of breast mass for 4 years and a diagnosis of IPC being delayed. The determination of IPC by physical examination and radiological imaging tends to be difficult, and early surgical intervention is prudent for a favorable outcome.

The exact etiology of male breast cancer is unknown. It seemed that IPC occurs more often in men with gynecomastia. Then it is speculated that imbalance in the estrogen-testosterone ratio has been implicated as risk factors. However, this relationship between gynecomastia and subsequent development of IPC is controversial, given the low incidence of male breast cancer and the broad prevalence of gynecomastia.^[8]^ We report the cases of 3 males without gynecomastia previously diagnosed. Serum tumor marker levels were all within normal limits. The blood estradiol, estriol, follicle-stimulating hormone, luteinizing hormone, testosterone, progesterone, and prolactin levels were normal.

It is of difficulty to differentiate IPC from benign disease due to the absence of significant symptoms. Clinical manifestations of IPC are not specific. Patients with IPC can be asymptomatic. Most patients with IPC of the breast have symptoms such as a palpable subareolar painless mass or nipple discharge. All patients presented with a palpable mass in our cases. Light brown nipple discharge occurred in 1 case. In addition, the mammographic appearance of IPCs resemble breast fibroadenoma. In the present study, IPC is often presented as a well-circumscribed, high-density mass without microcalcifications in the subareolar region. The margins of the mass are usually circumscribed.^[9]^

On ultrasound, the typical sonographic appearance of IPC may appear as a solid hypoechoic mass or a complex mass with cystic and nodular solid components with posterior acoustic enhancement.^[10]^ CEUS may be useful to diagnose IPC. An intact enhancing cyst wall was more commonly seen in malignant lesions compared to benign papillomas. Among 13 intracystic mass lesions, the cyst wall was wholly enhanced in all of 8 malignant papillomas lesions.^[11]^ CEUS is strongly suggested for further evaluation when a complex mass is detected on two-dimensional ultrasound.

If some CEUS features such as non-confluent enhancement, late overall wash-out, regional perfusion defect, early clumped shape, and penetrating vessels are identified in a male patient, an IPC should be considered, and ultrasound-guided core-needle biopsy or surgical excision might be necessary.^[11]^

It was challenging to distinguish IPC from benign papillomas, and papillary carcinoma by fine-needle aspiration as the rate of false negatives is about 22.0% to 37.8%.^[12]^ Four out of 9 patients were positive for malignant cells after undergoing fine-needle aspiration.^[13]^ Therefore, core-needle biopsy or surgical excision is essential for confirming the diagnosis of IPC, especially in older men.

Histologic diagnosis was based on the finding of a solitary circumscribed tumor with papillary, cribriform, and/or solid patterns of homogeneous, uniform cells with the absence of myoepithelial layer.^[14]^ IPC had been divided into 3 subsets: pure IPC, IPC associated with ductal carcinoma in situ (DCIS), and IPC associated with invasive cancer.^[15]^ Grabowski et al reviewed 917 cases of IPC and invasive carcinoma reported to occur in about 53% of cases.^[5]^ IPC is usually of low or intermediate nuclear grade, with no evidence of necrosis, strongly estrogen receptor-positive and negative for c-erbB-2.^[16]^

The mainstay of treatment is surgery, and there is no consensus for the surgical procedures of IPC. Patients were treated with mastectomy, or breast-conserving surgery can be plausible.^[7]^ Golshan et al reported no local recurrence in 7 male breast carcinoma treated with breast conservation.^[17]^

Surgical management of axillary for IPC patients is still controversial. Generally, axillary surgery is not recommended in patients with IPC and associated DCIS for its low frequency of axillary node metastases.^[2]^ However, an axillary staging is supported by some authors for axillary lymph node involvement is as high as 14% of the cases with IPC.^[2]^ Grabowski recommends treating IPC similar to DCIS with microinvasion, as 7.8% of the cases were classified as a regional disease.^[5]^ Then sentinel node biopsy may be considered in patients with IPC associated with DCIS or invasive carcinoma.^[9]^ Axillary sentinel lymph node biopsy was performed on 2 patients in our study, and no metastasis was identified in the sentinel lymph node. As a result, sentinel lymph node biopsy is a safe surgical treatment with minor injury for IPC patients from our experiences, and this procedure is worth considering.

There are no guidelines for the adjuvant therapy of IPC because of its rarity. Chemotherapy is usually unessential in IPC alone or with associated DCIS, and chemotherapeutic intervention may be considered when axillary lymph node metastasis present.^[18]^

The role of radiotherapy for the patients of IPC remains undefined. 30% of the patients in Solorazanos IPC series received adjuvant radiotherapy. The authors reported that the addition of radiation did not influence recurrence or survival.^[19]^ Chemotherapy or radiotherapy was not given in our cases.

Though over 90% of male IPCs are estrogen receptor-positive tumors.^[20]^ Grabowski et al confirmed that the addition of hormonal treatment does not appear to impact the outcome.^[5]^ All cases were estrogen receptor-positive in our circumstances. Two patients received adjuvant tamoxifen, and the other 1 was not given endocrine therapy. All cases have exhibited favorable prognosis by now, and long-term follow-up is indispensable to testify the efficacy of hormonal treatment.

In general, Patients with IPCs have excellent prognoses because of the low malignant potential. IPCs have reported a relative cumulative survival of 96.8% in patients with DCIS, whereas those with invasive IPC had a relative cumulative survival of 94.4% at 10 years.^[5]^

Although 1 IPC patients with a small focus of invasive carcinoma in our study, all the patients are disease-free during a median follow-up of 67 months (range, 13–120) months.

Our study has several strengths and limitations. In this report, to the best of our knowledge, we present the first case of IPC of the breast in man on CEUS. We described some CEUS features of IPC such as a rapid wash-in, slow wash-out with heterogeneous hyperenhancement, and regional perfusion defect, which may be useful to diagnose IPC prior to the operation. Limitations of this case reports included the small number of patients with only 3 cases, and only 1 patient performed CEUS, all of which limited our ability to summarize the typical sonographic appearance of IPC on CEUS. Another weakness of the study is that no magnetic resonance imaging (MRI) was performed to compare the enhancing features of IPC between MRI and CEUS.

## 4 Conclusions

Intracystic papillary breast carcinoma is extremely rare in males. Its slow growth and excellent prognosis generally characterize it. Clinical and mammographic manifestations of IPC are not specific. A solid hypoechoic mass or a complex mass with cystic and nodular solid components on ultrasound may be suspicious of IPC. Our results suggest that a rapid wash-in, slow wash-out with heterogeneous hyperenhancement is a characteristic appearance of papillary lesions on CEUS and can be useful in diagnosis. Surgical excision is the mainstay of the treatment, and the use of adjuvant therapy, hormonal therapy, and radiotherapy remain unclear.

## Author contributions

**Conceptualization:** Junling He, Haibin Xu.

**Data curation:** Hua Luo.

**Formal analysis:** Kexin Meng.

**Investigation:** Zujian Hu.

**Methodology:** Chenni Zhan.

**Project administration:** Kunlun Su.

**Resources:** Huifen Yang.

**Software:** Ouou Yang.

**Validation:** Tian Lan.
